# Supplementary material for: The Effectiveness of a CrossFit Training Program for Improving Physical Fitness of Young Judokas: A Pilot Study
Source: J Funct Morphol Kinesiol. 2022 Oct 8;7(4):83. doi: 10.3390/jfmk7040083 (PMC9590037; doi:10.3390/jfmk7040083)
Supplement: Supplementary file 1 [file jfmk-07-00083-s001.zip › jfmk-1882251-supplementary.pdf]

Supplementary Table S1. Sociological survey questions and answers (translated from Armenian).

N	Question	Possible answers
1	Do you enjoy participating in training?	1. Yes.<br>2. No.<br>3. Sometimes yes.<br>4. Sometimes no.<br>5. I find it difficult to answer.
2	Which method do you prefer most during CrossFit- training (you can mention several options):	1. Tabata.<br>2. AMRAP.<br>3. 21-15-9.<br>4. WOD.
3	What exercises do you most enjoy doing in CrossFit-training (you can mention several options):	1. Push-ups.<br>2. Squats.<br>3. Sit-ups.<br>4. Jumping exercises.<br>5. Static exercises.<br>6. Exercises with weight.
4	What types of exercises do you prefer (you can mention several options):	1. With your own body weight.<br>2. With partner.<br>3. With additional weights.
5	Do you have bad feelings during CrossFit training?	1. Yes.<br>2. No.<br>3. Rarely.
6	If you have bad feelings during training, mention them (you can mention several options):	1. Headache.<br>2. Nausea.<br>3. Dizziness.<br>4. Weakness.<br>5. Muscle cramps.
7	During which trainings do you mostly have the above-mentioned complaints (you can mention several options):	1. Tabata.<br>2. AMRAP.<br>3. 21-15-9.<br>4. WOD.<br>5. I have no complaints.
8	Do you find it difficult to do CrossFit exercises?	1. Yes, often.<br>2. Not often.<br>3. Sometimes.<br>4. I don't find it difficult.
9	What would you like to change in the content and performance of crossfit training (you can name 3 options):	1. Increasing the variety of exercises.<br>2. Increasing the number of repetitions.<br>3. Reducing the number of repetitions.<br>4. Increasing the duration of rest between exercises.<br>5. Reducing the duration of rest between exercises.<br>6. Nothing needs to be changed.
